# Activation of immune pathways in common bed bugs, *Cimex lectularius*, in response to bacterial immune challenges - a transcriptomics analysis

**DOI:** 10.3389/fimmu.2024.1384193

**Published:** 2024-04-17

**Authors:** Sanam Meraj, Nicolas Salcedo-Porras, Carl Lowenberger, Gerhard Gries

**Affiliations:** Department of Biological Sciences, Simon Fraser University, Burnaby, BC, Canada

**Keywords:** bed bugs, immune transcriptomics, antimicrobial peptides, toll & Imd pathways, vectors & pathogens, immunization, innate immunity

## Abstract

The common bed bug, *Cimex lectularius*, is an urban pest of global health significance, severely affecting the physical and mental health of humans. In contrast to most other blood-feeding arthropods, bed bugs are not major vectors of pathogens, but the underlying mechanisms for this phenomenon are largely unexplored. Here, we present the first transcriptomics study of bed bugs in response to immune challenges. To study transcriptional variations in bed bugs following ingestion of bacteria, we extracted and processed mRNA from body tissues of adult male bed bugs after ingestion of sterile blood or blood containing the Gram-positive (Gr+) bacterium *Bacillus subtilis* or the Gram-negative (Gr–) bacterium *Escherichia coli.* We analyzed mRNA from the bed bugs’ midgut (the primary tissue involved in blood ingestion) and from the rest of their bodies (RoB; body minus head and midgut tissues). We show that the midgut exhibits a stronger immune response to ingestion of bacteria than the RoB, as indicated by the expression of genes encoding antimicrobial peptides (AMPs). Both the Toll and Imd signaling pathways, associated with immune responses, were highly activated by the ingestion of bacteria. Bacterial infection in bed bugs further provides evidence for metabolic reconfiguration and resource allocation in the bed bugs’ midgut and RoB to promote production of AMPs. Our data suggest that infection with particular pathogens in bed bugs may be associated with altered metabolic pathways within the midgut and RoB that favors immune responses. We further show that multiple established cellular immune responses are preserved and are activated by the presence of specific pathogens. Our study provides a greater understanding of nuances in the immune responses of bed bugs towards pathogens that ultimately might contribute to novel bed bug control tactics.

## 1 Introduction

Evolutionary biology studies revealed that genes associated with the canonical immune system often evolve most rapidly, both within and between species (1, 2). With the growing number of insect genomes and transcriptomes being sequenced, it has become evident that duplication and loss of immune genes are likely caused by host-pathogen interactions (1, 2). Moreover, certain species react to various pathogenic challenges with a diverse array of specific responses (see below). RNA-seq has become a crucial technique to detail the infection-modulated transcriptome of various insects. Such studies significantly contribute to our understanding of the complex interplay between ecology and evolution in host response mechanisms by revealing dynamic gene expression changes involved in host responses to environmental variations and stresses. Immune transcriptomes or transcription of immune genes in several pathogen-challenged hematophagous insects, such as mosquitoes, sand flies, and kissing bugs, have been reported (3–11), but the transcriptome of pathogen-challenged common bed bugs, *Cimex lectularius*, is still not known despite their global health significance (12–21). The present study aims to address this gap by focusing on the transcriptome of common bed bugs in response to infection with bacteria.

Common bed bugs are obligate hematophagous ectoparasites that primarily feed on humans but also parasitize bats, birds, and other vertebrates (14, 22, 23). Bed bug bites commonly cause various degrees of cutaneous and systemic reactions in humans (24), and bed bug infestations greatly affect human mental health (25). Being wingless with limited dispersal ability, bed bugs largely rely on vertebrate hosts for transport to new dwellings (26). In recent decades, many countries have experienced a resurgence of bed bug infestations (16, 17, 27, 28), with global bed bug populations estimated to increase annually by 100–500% (20, 29).

Although more than 40 potential disease-causing agents (bacteria, viruses, parasites) have been detected in or on bed bugs (19), and bed bug infestations are strongly correlated with the occurrence of various human illnesses (30), bed bugs are not known to vector these parasites and pathogens to humans outside laboratory settings (31). The underlying physiological and immune mechanisms responsible for the bed bugs’ vector incompetence are not well understood, hence the need to study the range and anti-parasite activity of innate immune factors in bed bugs on their vector potential.

Like other invertebrates, insects have no adaptive immune system (32–35). Instead, they cope with pathogen infections through innate immune responses that include complex general- and pathogen-specific signaling pathways. Some evolutionarily conserved pathways are common to all insects, but there are also species-specific evolutionary adaptations, such as the expansion or contraction of immune gene families (36–39), possibly in response to selective pressure exerted by pathogens and environments (36–39). In insects, the recognition of non-self initiates innate immune cascades, triggering – among others – phagocytosis, melanotic encapsulation, and the expression of antimicrobial peptides (AMPs) (40–42).

The best characterized molecular pathways involved in innate immune responses in insects are the Imd (4, 43, 44), Toll (3, 40, 44), and Janus-kinase (45) signaling pathways. Initially, these pathways were thought to be activated by distinct classes of pathogens: the Toll pathway by Gram-positive (Gr+) bacteria and fungi, the Imd pathway by Gram-negative (Gr–) bacteria, and the Janus-kinase pathway by bacteria, viruses, and parasites (45). It is now accepted that there is significant plasticity and cross-talk among multiple immune pathways (44).

The Imd signaling cascade is activated when peptidoglycan recognition proteins (PGRPs) recognize pathogen-associated molecular patterns (PAMPs) such as the diaminopimelic acid-type peptidoglycan (PGN) in the cell walls of Gr– bacteria (4), resulting in the translocation of the NF-κB transcription factor *relish* from the cytoplasm to the nucleus, and the subsequent production of AMPs and other effectors (e.g., reactive oxygen species, lysozymes, protease inhibitors) which provide systemic innate immunity (4).

The Toll pathway is initiated through both the recognition of pathogen-associated molecular patterns (PAMPs), by pattern recognition receptors (PRRs), and by a cascade of serine proteases (SPs), with serine protease inhibitors modulating SPs and thus regulating signal amplification (3, 40, 42, 44, 46). In *Drosophila melanogaster*, PAMPs are mainly the lysine-type PGN of Gr+ bacteria, and the β-1,3 glucans of fungi. These recognition events activate the Toll-receptor ligand *Spätzle* which – together with the proteins Myd88, tubulin-ϵ, and Pelle – forms a protein complex whose function is the degradation of the Cactus gene and, ultimately, the translocation of transcription factors into the nucleus, and the production of AMPs (42, 47, 48).

The Janus kinase/signal transducer and activator of the transcription JAK/STAT pathway coordinate immune responses from cytokines and regulate multiple homeostasis mechanisms in hosts (45). The Janus-kinase pathway responds to infections and is implicated in regulations of cell growth, differentiation and apoptosis, as well as in inflammatory reactions (45).

The fat body is the principal immune organ in insects, and is also a major metabolic organ, storing and providing energy for the entire organism. During infections, there is an internal switch in the fat body from anabolism to the production of antimicrobial peptides (49–51). Activation of an immune response prompts a rapid metabolic reconfiguration that may be energetically intensive, characterized by increased ATP production and a higher demand for specific nutrients such as glucose and glutamine, facilitating rapid antimicrobial peptide synthesis (51). Organismal resources may be preferentially redirected toward critical immunological functions, often at the expense of other physiological processes, such as growth and reproduction, to combat pathogens effectively (50, 51). This delicate metabolic balancing act ensures that energy allocation is optimized for immune defense while minimizing detrimental effects on overall fitness. In this context, the metabolic changes in the host are associated with the induction of innate immune processes such as the synthesis of AMPs and the mobilization of immune cells (51). These metabolic changes attempt to eliminate pathogens or to minimize the impact of infection.

Common bed bugs possess genes representative of the Imd, Toll, and Janus-kinase pathways. Their gene repertoire for some pathway components, especially those of the Imd pathway, is reduced compared with holometabolous insects (22), and similar to what has been reported in some other hemimetabolous insects (22, 35). Bed bugs are known to produce defensins and prolixicins as major AMPs in response to bacterial infection (Meraj et al., 2022; unpubl. data), but immune pathway changes in bed bugs, as well as metabolic changes of their midgut and fat body, have not yet been explored.

Here, we present a transcriptomic analysis of common bed bugs in response to bacterial infections. We focus on the bed bugs’ innate immune responses, particularly the Imd and Toll pathways, which are instrumental in the production of AMPs. Our findings add to the growing body of literature and knowledge about vector insect immunology, and may contribute to the development of new tactics that address the challenges posed by bed bugs and other hematophagous insects.

## 2 Materials and methods

### 2.1 Collection and rearing of bed bugs

For a start-up colony, bed bugs were supplied by Harold Harlan (Crownsville, MD, USA) and were collected in infested apartments in Vancouver (BC, Canada). The bed bug colony was maintained as previously described (52). Briefly, bed bugs were kept in the insectary of Simon Fraser University (SFU) at a temperature of ∼24°C, ambient relative humidity, and a photoperiod of 14 h light to 10 h dark. Groups of 150 bed bugs were maintained in 50-mL glass jars fitted with a square of cardboard (2 cm × 2 cm) at the bottom and a strip of cardboard (2 cm × 4 cm) diagonally across the jar. Bed bugs in separate jars were fed on the forearm of a volunteer (Regine Gries) once every month. For feeding, jars were covered with fine mesh, inverted, and pressed against the forearm so that the bed bugs could feed through the mesh.

### 2.2 Growth and preparation of bacteria and immune challenges of bed bugs

Live bacteria (instead of isolated lipopolysaccharides, lipoteichoic acid, or peptidoglycans) were used to simultaneously elicit multiple immune responses. We selected *Escherichia coli* (K12/D31) and *Bacillus subtilis* subsp. *spizizenii* (ATCC 6633) because of their well-characterized immune-stimulatory properties and to establish a baseline understanding of bed bug immune responses. Future studies will then focus on pathogenic bacteria.

*Escherichia coli* and *B. subtilis* were grown in separate Lysogeny Broth (LB) (53) for 17 h at 30°C in a shaking incubator (220 revolutions per minute). Subsequently, aliquots of bacterial broth were reinoculated into fresh broth and incubated 4 h under the same conditions to reach the log phase of growth. The bacteria were then washed three times in sterile phosphate buffered saline (PBS; 0.01 M phosphate buffer, 2.7 mM potassium chloride, 0.137 M sodium chloride, pH 7.4), and were diluted in sterile defibrinated rabbit blood (void of viruses and endotoxins; Hemostat, Dixon, CA, USA) to a final concentration of ~1 × 10^6^ cells/mL. Male adult bed bugs (food-deprived >20 days) fed 1 h on defibrinated, microbe-laced (*E. coli* or *B. subtilis*) rabbit blood in a water-jacketed membrane feeder (Thermo Fisher Scientific Isotemp 2150 B14, USA) set to 37°C, with stretched out parafilm as the membrane. Control bed bugs ingested sterile blood. As both female and male immune-challenged bed bugs upregulate their immune responses, we worked with males because they pose no risk of starting a new infestation in case of their accidental escape from laboratory confinements. Fully engorged bed bugs were separated and housed in glass jars until analysis. For tissue isolation, RNA extraction and transcriptome assembly (see below), we used three (out of five) top-quality replicates, with each replicate containing five insects in treatment and control samples.

### 2.3 Tissue isolation and RNA extraction

Midgut and RoB tissues (RoB = rest of the body: bodies without heads and midgut tissues) of treatment and control adult male bed bugs were dissected by hand (using fine dissection tools) 12 h after an immune challenge, and total RNA was extracted using TRizol reagent (Invitrogen) following the manufacturer’s recommendations. Midguts of bed bugs were washed three times in PCR before RNA extraction. RNA quantity and quality were assessed using Nanodrop 1000 spectrophotometer v. 3.7 (Thermo Fisher Scientific, USA), Qubit Fluorometer (Thermo Fisher Scientific, USA), gel electrophoresis, RNA ScreenTape assay (Agilent, USA), and RT-qPCR with RPL18 (internal control) (54) and CL-defensin3. Only RNA that passed this rigorous quality assessment was used for transcriptome assembly.

### 2.4 Transcriptome assembly: library preparation with polyA selection, HiSeq sequencing, and RNA-Seq data analyses

We created a *de novo* transcriptome assembly from RNA extracted from midgut and RoB tissues of male bed bugs 12 h after ingesting blood containing *E. coli* or *B. subtilis*. RNA purification, first and second strand synthesis, adaptor ligation, quantification, validation, and Illumina sequencing were all done at GENEWIZ LLC. (South Plainfield, NJ, USA).

The RNA samples were sequenced using a paired-end (PE) configuration, consisting of two 150 base pair (bp) strands. HiSeq Control Software (HCS) was used for image analysis and base calling. Raw sequence data (.bcl files) generated from Illumina HiSeq were converted into fastq files and demultiplexed with bcl2fastq 2.17 software (Illumina Inc, San Diego, CA, USA). For index sequence identification, one mismatch was allowed. After examining the quality of raw data, adapter sequences and nucleotides of poor quality were removed from sequence reads using Trimmomatic v.0.36. The trimmed reads were mapped to the reference genome available on ENSEMBL, using STAR aligner v.2.5.2b which utilizes a splice aligner to detect splice junctions and incorporates them to help align the entire read sequences to generate Binary Alignment Map (BAM) files. In the Subread package version 1.5.2, we used the ‘Counts’ feature to calculate the unique gene count. Only reads predicted to fall within exons were counted.

We used the gene hit counts table for downstream differential expression (DE) analysis, using DESeq2 (55) to compare gene expression among groups of samples. We used the Wald’s test to generate *p*-values and Log2 Fold Changes (Log2FC). We considered genes to be differentially expressed (DE) when adjusted *p*-values were < 0.05 and the overall absolute Log2FC was > 1. The data for immune transcripts were mined to evaluate their expression levels, and are reported as transcripts per million (TPM). Additional transcriptome studies on *C. lectularius* are accessible on VectorBase (https://vectorbase.org/vectorbase/app/search?q=cimex), providing a valuable resource for future comparative analysis.

### 2.5 Bioinformatic analyses

All analyses were performed in R v.4.2.1 (56) using the Rstudio environment v. 2022.12.0 Build 353. The following packages were used: *DESeq2* (57)*, ggplot2* (58), *tidyverse* (59)*, EnhancedVolcano* (60), *clusterProfiler* (61), pathview (62)*, ggvenn* (63), *pheatmap* (64), and *RcolorBrewer* (65). Gene orthologues to known *D. melanogaster* genes were found using the R package *orthogene* (66). Immune-related genes were named after Benoit et al. (2016). Gene Ontology (GO, http://geneontology.org/) enrichment and Kyoto Encyclopedia of Genes and Genomes (KEGG, http://www.kegg.jp/) pathway analyses were performed using the R package *clusterProfiler* (61). Results with *P* < 0.05 were considered statistically significant.

## 3 Results and discussion

To study transcriptional variations in common bed bugs following ingestion of bacteria, we extracted and processed mRNA from adult male bed bugs after ingestion of sterile blood or bacteria-laced blood. Samples obtained after bed bugs ingested sterile blood are referred to as ‘midgut/control’ or ‘RoB/control’, whereas samples obtained after bed bugs ingested blood laced with Gr+ or Gr– bacteria are referred as ‘midgut/Gr+’, ‘midgut/Gr–’, ‘RoB/Gr+’, and ‘RoB/Gr–’.

As many as 18 transcriptomes were obtained, with a mean number of reads of 18,232,331.7. Of these reads, 15,100,243 (83%) were mapped on the *C. lectularius* reference genome, and 14,889,639 (81.6%) were unique mapped reads (**Supplementary Table S1**). In the assembled transcriptome, we found 11,558 unique genes. Of these genes, 8,422 were differentially expressed (DE) (p<0.05) in at least one of the pair-wise comparative analyses (**Supplementary Table S2**). By comparing the expression of transcripts and possible splice-variants, we were able to discriminate among genes and gene isoforms with similar sequences, which we report as single genes with multiple isoforms in our assembly pipeline.

### 3.1 Differential expression patterns

**Figure 1:**
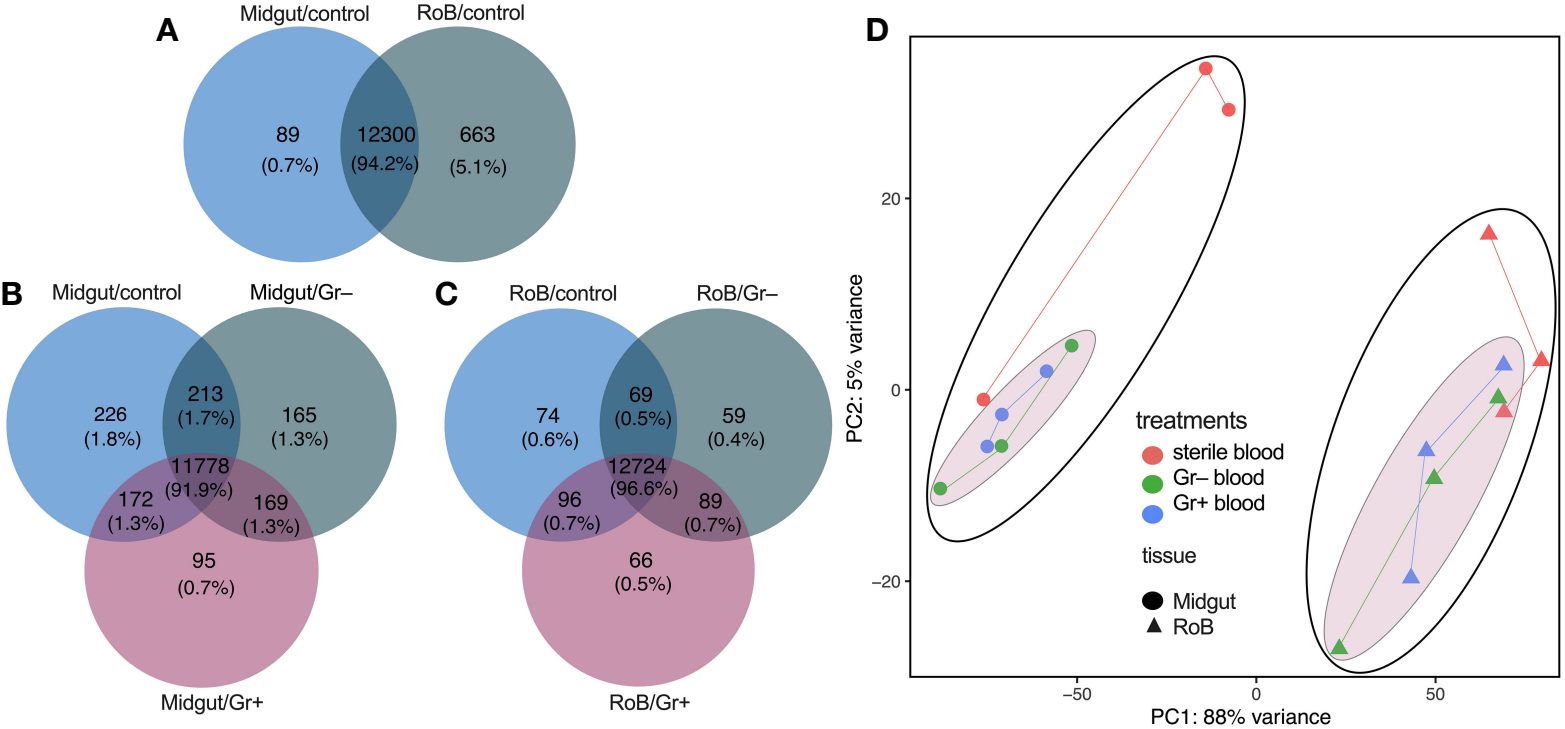
Venn diagrams and principal component analysis of treatment-dependent differentially expressed (DE) transcripts in two tissue types of bed bugs. **(A–C)** DE transcripts in midgut and rest of body (RoB: body minus head and midgut tissue) samples obtained from adult male bed bugs after ingestion of sterile blood (control treatment) **(A)** or blood laced with the Gram-negative (Gr–) bacterium *Escherichia coli* K12/D31 or the Gram-positive (Gr+) bacterium *Bacillus subtilis* subsp. *spizizenii* ATCC 6633 **(B, C)**. Absolute numbers (and percentages of total transcripts) inside circles, and circle overlaps, represent the numbers (and percentages) of transcripts expressed exclusively in that circle, or circle overlap. **(D)** Principal component analysis (PCA) plots demonstrating treatment effects in midgut and RoB tissues. Expression levels were used to inform the PCA.

The set of genes expressed in midgut and RoB samples of male bed bugs was strikingly similar regardless of tissue type and experimental treatment, the ingestion of sterile or bacteria-laced blood. Among the three midgut sample types (midgut/control, midgut/Gr+, and midgut/Gr–), 91.9% of transcripts were shared (**Figure 1B**), with most (1.8%) of the non-shared transcripts being present in midgut/control samples. Among the three RoB sample types (RoB/control, RoB/Gr+, and RoB/Gr–), 96.6% of expressed genes were shared (**Figure 1C**). Sterile blood ingestion afforded similar effects in midgut and RoB samples, with 94.2% of transcripts being shared (**Figure 1A**), suggesting that these two tissue types have similar transcribed genes. In contrast, *levels* of gene expression markedly differed between midgut and RoB samples irrespective of treatment (**Figure 1D**), as revealed by principal component analysis (PCA), where the two main PCA axes explained 93% of variations. Control samples (obtained from bed bugs that ingested sterile blood) were better separated from treatment samples (obtained from bed bugs that ingested bacteria-laced blood) in midgut tissues than in RoB tissues (**Figure 1D**).

### 3.2 Differential expression analysis of top 30 genes across multiple comparisons

**Figure 2:**
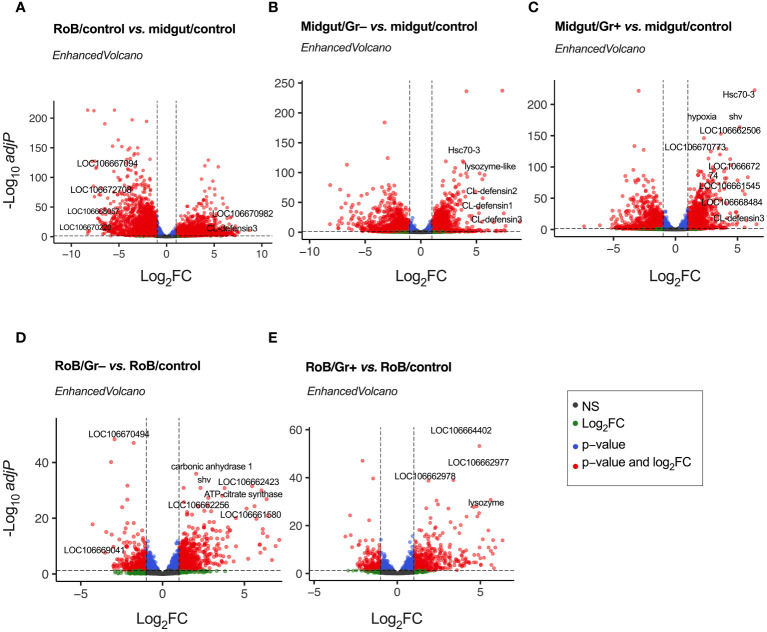
Volcano plots of treatment-dependent differentially expressed (DE) genes in bed bugs. Plots illustrate the global transcriptional change in pair-wise comparisons of midgut and rest of body (RoB: body minus head and midgut tissues) samples obtained from adult male bed bugs obtained after ingestion of sterile blood (control treatment) or blood laced with the Gram-negative (Gr–) bacterium *Escherichia coli* K12/D31 or the Gram-positive (Gr+) bacterium *Bacillus subtilis* subsp. *spizizenii* ATCC 6633. All genes are plotted and each data point represents one gene. The log2 Fold Change (log_2_FC) of each gene is presented on the x-axis, and the log10 of its adjusted p-value is presented on the y-axis. Red points above the dotted x-axis represent genes (some of which annotated) that are significantly up- or down-regulated with an adjusted p value (padj) < 0.05 and a Log_2_ Fold Change (log_2_FC) > |1|. Blue dots represent genes with padj value < 0.05 and log_2_FC < |1|. Green dots represent non-significant genes with log_2_FC > |1|, and grey dots represent non-significant genes with log_2_FC < |1|.

Genes with an adjusted *p*-value of <0.05, and an overall absolute log2FC >1, were considered DE genes for all comparisons, and are listed in **Supplementary Table S2**. **Figure 2** and **Supplementary Table S3** display volcano plots for significant DE genes in various pairwise comparisons. Top-30 highly up-regulated genes in each of the comparisons are discussed below.

#### 3.2.1 Midgut *vs*. RoB: responses to ingestion of sterile blood

Differential gene expressions in midgut/control and RoB/control samples were analyzed to unveil distinct molecular responses of these tissues after bed bugs ingested sterile blood (**Figure 2A**; **Supplementary Tables S2, S3**). Following normalization, 5234 DE genes were identified (**Supplementary Table S1**). “Up-regulated” genes exhibited higher expression in the RoB control than in the midgut control, whereas “down-regulated” genes had reduced expression in the RoB control relative to the midgut control (**Figure 2A**). Up-regulated genes in the midgut were genes encoding transport mechanisms (67), such as the zinc transporter ZIP3-like gene (LOC106667094) and the facilitated trehalose transporter Tret1-like gene (LOC106672708). Some genes with enzymatic functions also stood out, such as the (probable) cytochrome P450 6a13 gene (LOC106670220) and the aspartic proteinase A2-like gene (LOC106668057). Conversely, up-regulated in RoB were genes encoding (*i*) RNA binding such as the RNA-binding motif protein X-linked-like-3 gene (LOC106666697), (*ii*) cuticle formation, such as the hornerin-like gene (LOC112127793), and (*iii*) metabolic processes such as the glucose dehydrogenase FAD, quinone-like gene (LOC106670982). Among immune genes, CL-Defensin3 was also up-regulated in RoB/control compared to midgut/control. Among all the up- and down-regulated genes, many were not characterized.

#### 3.2.2 Midgut responses to ingestion of bacteria

Ingestion of bacteria prompted a marked shift in gene expression in the midgut, underscoring its defensive role in mitigating bacterial challenges. Analyses of differential gene expression between midgut/control and midgut/Gr– samples revealed 3152 DE genes (**Figure 2B**; **Supplementary Tables S1, S3**). Among these, defense- and immunity-associated genes were prominently up-regulated. Key genes include CL-defensin3 (LOC106661791), CL-defensin2 (LOC106661792), and CL-defensin1 (LOC106661793), which are modulated by the Imd and Toll pathways (68, 69). The lysozyme-like gene (LOC106663700) aligns with the c-type lysozymes RpLys-A (EU250274) and RpLys-B (EU250275) from *Rhodnius prolixus* (70) that function in both digestion and immune defense by targeting bacterial cell walls (70). The heat shock protein gene Hsc70-3 (LOC106669548) not only provides protection against thermal stress from blood ingestion but also plays a pivotal role in digestion-related signaling pathways (71, 72). The genes mentioned here merely serve as examples, with a comprehensive list of 30 highly DE genes being reported in **Supplementary Table S3**. Notably, during bacterial encounters, a significant number of genes is down-regulated. Whether this is due to strategic metabolic adjustments by the insect for energy conservation and resource reallocation (**Supplementary Table S3**), or initiated by pathogens to reduce, eliminate, or circumvent host immune responses is not clear.

Analyses of differential gene expression between midgut/control and midgut/Gr+ samples identified 3162 DE genes (**Figure 2C**; **Supplementary Tables S2, S3**), about as many DE genes as we found in analyses of differential gene expression between midgut/control and midgut/Gr– samples (see above). Among the prominently up-regulated genes were again defensins, Hsc70-3, and venom serine carboxypeptidase-like genes (LOC106668484), all of which are vital components of innate immunity (33, 68, 69, 73–75). Concurrently, genes were accentuated that are integral to transport mechanisms, such as the sodium-dependent dopamine transporter gene (LOC106661545) and the acetyl-coenzyme A transporter 1 gene (LOC106662506). Activated cellular stress responses were reflected in enhanced expressions of genes associated with stress and protein folding, such as the dnaJ homolog shv gene (LOC106666782) and the endoplasmin gene (LOC106670773). Shifting metabolic dynamics were indicated by up-regulation of the hypoxia protein1 gene (LOC106670186) and the fatty acid synthase-like gene (LOC106667274). In contrast, genes associated with processes such as synaptic vesicle exocytosis, immune responses, and environmental stress adaptation were down-regulated (see **Supplementary Table S3** for details). In summary, these changes in gene expression by bed bugs upon bacterial exposure suggest a functional role of the midgut in adapting and adjusting immune responses.

#### 3.2.3 RoB Responses to bacteria ingestion

Analyses of differential gene expressions in RoB/control and RoB/Gr– samples revealed 997 DE genes (**Figure 2D**; **Supplementary Tables S2, S3**). Highly up-regulated were genes integral to metabolic processes, such as the carbonic anhydrase 1 gene (LOC106661111) and the ATP-citrate synthase gene (LOC106665077), as well as genes involved in protein regulation, such as the polypeptide N-acetyl-galactosaminyltransferase 5 gene (LOC106662256) and the dnaJ homolog shv gene (LOC106666782). Also up-regulated were cuticle formation genes including the laccase-5-like oxidoreductase gene (LOC106661580), and probable defense mechanism genes such as the senecionine N-oxygenase-like gene (LOC106662423). Down-regulated were genes involved in metabolism and energy production, such as the mitochondrial pyruvate carboxylase gene (LOC106669041), the L-threonine dehydratase catabolic TdcB gene (LOC106670494), and others (**Supplementary Table S3**).

Analyses of differential gene expressions in RoB/control and RoB/Gr+ samples revealed 654 DE genes (**Figure 2E**; **Supplementary Tables S2, S3**). Up-regulated genes are primarily associated with signal transduction, cuticle formation, and defense mechanisms. For example, tyrosine 3-monooxygenase (LOC106664402) plays a critical role in the production of catecholamines which are crucial for signal transduction and melanogenesis. Enzymes such as inositol polyphosphate 5-phosphatase (LOC106662977) and inositol polyphosphate 5-phosphatase K (LOC106662978) are implicated in phosphoinositide metabolism, which is crucial for intracellular signaling and trafficking. Acetylcholinesterase (LOC106669436 & LOC112127332) regulates synaptic transmission by terminating synaptic signals. The upregulation of lysozyme (LOC106666694), with 30% similarity to c-type lysozymes RpLys-A (EU250274) and RpLys-B (EU250275) from *R. prolixus* (70), suggests an immune-related function of this lysozyme. Other up-regulated genes were those involved in cuticle formation such as cuticle protein genes (LOC106665132, LOC106663240, LOC106665305, LOC106664300, LOC106664954, LOC106664983, LOC106663020, LOC106663472, **Supplementary Table S3**), the fibroin heavy chain-like gene (LOC106661923), and the endocuticle structural glycoprotein SgAbd-2-like gene (LOC106665194). Conversely, down-regulated genes indicate decreased metabolism and detoxification (**Supplementary Table S3**).

Notably, in all functional categories many DE genes were not characterized (**Supplementary Table S3**). Further significantly up- or down-regulated genes in other pair-wise sample comparisons (midgut/Gr+ *vs.* midgut/Gr–; RoB/Gr+ *vs.* RoB/Gr–; midgut/Gr+ *vs.* RoB/Gr+; midgut/Gr– *vs.* RoB/Gr–) are listed in **Supplementary Table S2**.

In response to pathogens that have breached or damaged barriers, insects rapidly activate immune signaling pathways at both local (tissue/organ) and systemic (hemocoel) levels to clear infections (73, 76). Local and systemic immune responses, and molecular communication between different immune organs/tissues, are well documented in *Drosophila* spp. as well as *Anopheles* spp. and *Aedes* spp. (77, 78). For example, oral ingestion of bacteria (local challenge) by *Ae. aegypti* significantly modulated AMP expression in both the midgut and fat body (77, 78). Whereas it is not likely that ingested bacteria have entered the bed bugs’ hemocoel and interacted directly with tissues in the hemocoel, our observed variations in gene expression resemble those reported in vinegar flies and mosquitoes (77, 78).

### 3.3 Differential expression of immune-related genes

The number of immune-related DE genes found in pairwise sample comparisons are summarized in **Table 1**, and all immune-related DE genes are reported in **Supplementary Table S4**. Most DE genes, particularly up-regulated DE genes, were found in pairwise comparisons of midgut and RoB samples (**Table 1**; bottom three rows). Numbers and proportions of DE genes in control sample comparisons (RoB/control *vs.* midgut/control) differed from those of treatment *vs.* control sample comparisons. Particularly, in sample comparisons of RoB/control and midgut/Gr+, and RoB/control and midgut/Gr–, only 7% and 13% of DE genes, respectively, were down-regulated (**Table 1**). To further elucidate the roles of DE genes in immunological responses, we focused on specific effector molecules and immune pathways.

**Table 1:** Numbers (#) of immune-related genes with significant differential expressions (adjusted *p*-value: *<0.05*, absolute log2 Fold Change: >1) in pair-wise comparisons.

Type of comparison^1,2^	# up-regulated genes	# down-regulated genes
Midgut/Gr– *vs.* midgut/control	39	27
Midgut/Gr+ *vs.* midgut/control	35	20
RoB/Gr– *vs.* midgut/control	28	4
RoB/Gr+ *vs.* midgut/control	27	2
RoB/control *vs.* midgut/control	74	31
RoB/Gr+ *vs.* midgut/Gr+	87	35
RoB/Gr– *vs.* midgut/Gr–	89	45

^1^Samples obtained after bed bugs ingested sterile blood are referred to as ‘midgut/control’ or ‘RoB/control’, whereas samples obtained after bed bugs ingested blood laced with Gr+ or Gr– bacteria are referred as ‘midgut/Gr+’, ‘midgut/Gr–’, ‘RoB/Gr+’, and ‘RoB/Gr–’

^2^RoB, Rest of body: including bodies without heads and midgut tissues.

#### 3.3.1 AMPs

**Figure 3:**
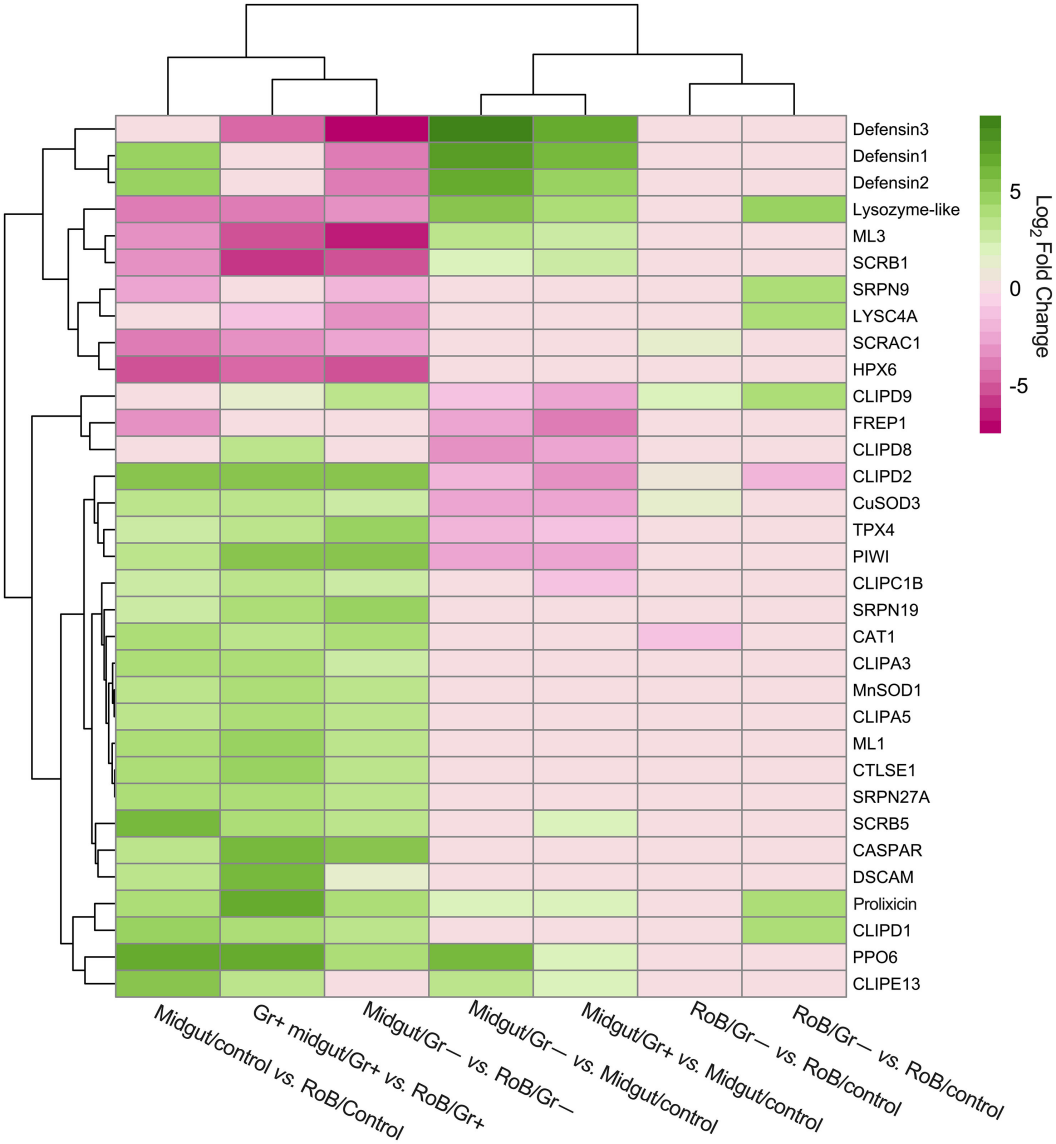
Treatment-dependent heatmap of most variable differentially expressed (DE) immune-related genes in bed bugs. Map data are based on six pair-wise comparisons of midgut and rest of body (RoB: body minus head and midgut tissue) samples obtained from adult male bed bugs after ingestion of sterile blood (control treatment) or blood laced with the Gram-negative (Gr–) bacterium *Escherichia coli* K12/D31 or the Gram-positive (Gr+) bacterium *Bacillus subtilis* subsp. *spizizenii* ATCC 6633. Genes are colored according to their Log_2_ Fold Change (see legend) and clustered using Euclidean distances.

**Figure 4:**
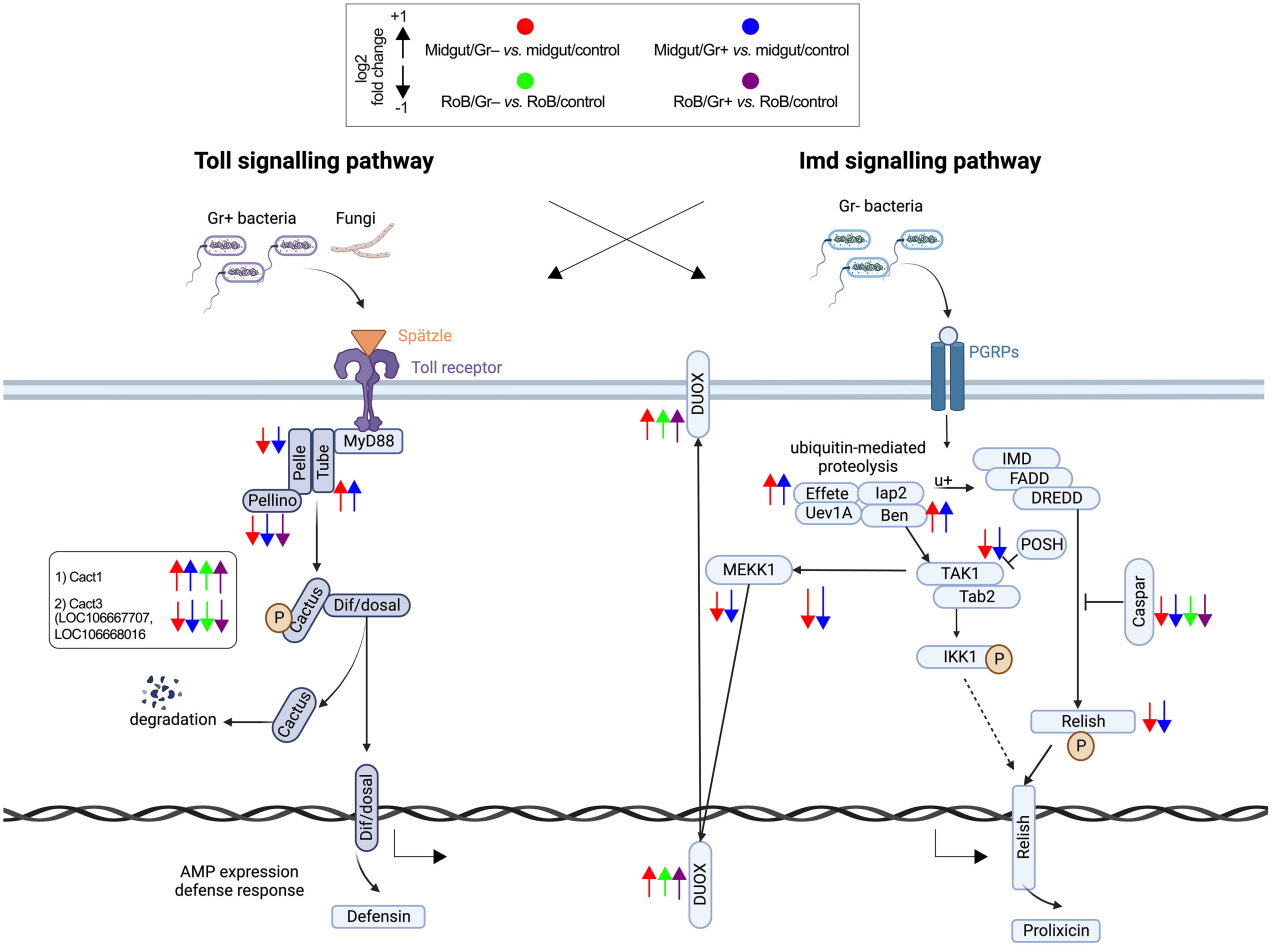
Treatment-dependent differential expression of Toll and Imd pathway components in bed bugs. Assignments of immunized components are based on data in pair-wise comparisons of midgut and rest of body (RoB: body minus head and midgut tissue) samples obtained from adult male bed bugs in response to ingestion of sterile blood (control treatment) or blood laced with the Gram-negative (Gr–) bacterium *Escherichia coli* K12/D31 and the Gram-positive (Gr+) bacterium *Bacillus subtilis* subsp. *spizizenii* ATCC 6633. Arrows pointing upward and downward indicate genes that are significantly up-regulated (log_2_ Fold Change >1) and down-regulated (log_2_ Fold Change < -1), respectively. Components in the midgut immunized by Gr– bacteria and Gr+ bacteria are marked by red and blue arrows, respectively.

In prior work, RT-qPCR revealed significant upregulation of CL-defensins and CL-prolixicins in response to ingestion of bacteria (69), supporting the results of this study. In summary, three defensin genes (CL-*defensins* 1, 2, and 3) were among the most up-regulated genes in pair-wise comparisons between midgut/controls and either midgut/Gr+ or midgut/Gr– samples (see 3.2; **Figures 3**, **4**; **Supplementary Table S4**). CL-defensins 1 and 2 in midgut/controls were up-regulated relative to RoB/controls, indicating that the expression of these genes in response to the ingestion of sterile blood differs in the two tissue types. Interestingly, the three CL-defensins were down-regulated in midgut/Gr– relative to RoB/Gr– samples. In this study, defensins and prolixicins were among the most highly up-regulated genes in the midgut, with defensins reaching a log2 fold expression level change of 9. Notably, this level surpasses that reported in comparable studies with kissing bugs, mosquitoes, and vinegar flies, where defensins did not appear to be as highly up-regulated (5, 6, 9, 70, 79–85). These different levels of expression warrant further exploration.

#### 3.3.2 Lysozymes

LYSC4A (LOC106663584, LOC106663588, LOC106669094, LOC106673043) and lysozyme-like molecules (LOC106663700, LOC106666694, LOC106667626) (see 3.2, **Figure 3**; **Supplementary Table S4**) were expressed at different levels in response to different treatments. In midgut/Gr– and midgut/Gr+ samples, the expression level of LOC106663700 increased (Log2FC) by 5.29 and 3.95, respectively. Only in RoB/Gr– samples was there a significant increase of almost all lysozyme transcripts (LOC106663584, LOC106663588, LOC106663700, LOC106666694, LOC106667626, LOC106669094). This variability indicates the multifaceted role that LOC106663700 may play in different body regions, with its expression being influenced by multiple factors.

#### 3.3.3 Prophenoloxidase

In midgut/Gr– samples, among the four PPO6 proteins (LOC106662258, LOC106673360, LOC106673564, LOC106673939) presented in **Supplementary Table S4**, only LOC106673360 was significantly up-regulated (Log2FC: 6.13). In midgut/Gr+ samples, only LOC106673564 (Log2FC: 2.28) and LOC106673939 (Log2FC: 1.84) were significantly up-regulated. In RoB tissues, there were no significant changes in PO transcription levels. This suggests that bacteria might be confined to, and possibly eliminated in, the midgut, preventing a PPO response in the RoB, and potentially precluding bacterial entry into the hemocoel.

#### 3.3.4 Recognition molecules

When we examined the expression of recognition molecules across treatments (**Figures 3**, **4**; **Supplementary Table S4**), several molecules showed significant changes in expression response to bacterial challenges. Specifically, in midgut/Gr– samples, CTLGA3 and CTL9 were significantly up-regulated (Log2FC of 2.60 and 1.66, respectively), whereas CTLGA1 was notably down-regulated (Log2FC -3.13). In RoB/Gr– samples, up-regulated molecules included CTLMA6 (Log2FC 2.22), CTLGA3 (Log2FC 3.96), and CTL9 (Log2FC 1.25). In RoB/G+ samples, CTLMA6 (Log2FC 1.80), CTLGA3 (Log2FC 3.47), and CTL9 (Log2FC 0.59) were significantly up-regulated, whereas CTLGA2 was down-regulated (Log2FC -1.28). Based on differential expressions in response to *E. coli* and *B. subtilis*, CTLGA3 and CTL9 appear to be broad-spectrum, likely interacting with both Toll and Imd pathways. Conversely, CTLGA1 and CTLGA2 may have pathway-specific roles, warranting further investigation.

#### 3.3.5 Activation of the Toll pathway following bacterial exposure

The Toll signaling pathway, a cornerstone of the insects’ innate immune responses that detect and respond to invading pathogens, becomes prominently activated during bacterial infections. Among the five Toll-related molecules (LOC106662834, LOC106670370, LOC106672311, LOC106672317, and LOC106672979) that primarily function as pattern recognition receptors and that we investigated (**Figures 3**, **4**; **Supplementary Table S4**), TOLL1 (LOC106670370) and TOLL10 (LOC106672311) were significantly up-regulated in both midgut/Gr+ and RoB/Gr+ samples. The amplified expression of these molecules underscores their pivotal roles in the heightened response of the Toll pathway when challenged with bacteria. Conversely, TAK1 (LOC106666550) – a kinase pivotal in transducing signals from Toll-like receptors to downstream effector mechanisms – was markedly down-regulated in midgut/Gr+ samples, suggesting potential mechanisms that might dampen Toll responses as needed. The cactus gene was up-regulated in midgut/Gr– relative to midgut/control samples. There were no DE genes in the Toll pathway when we compared the effects of Gr– and Gr+ in RoB samples.

#### 3.3.6 Modulation of the Imd pathway in response to bacterial exposure

The Imd pathway also plays a crucial role in insect immune responses by producing antimicrobial peptides (AMPs). Ingestion of Gr– and Gr+ bacteria by bed bugs caused differential effects in midgut samples for the Imd pathway (**Figure 3**, **4**; **Supplementary Table S4**). The Imd pathway, which traditionally has been considered as a defense against Gr– bacteria, revealed intriguing patterns in gene expression upon bacterial exposure (**Figures 3**, **4**; **Supplementary Table S4**). The REL2 gene (LOC106667016), a transcription factor central to the activation of the Imd pathway (3), was significantly up-regulated in both RoB/Gr+ and RoB/Gr– samples. As expected in Imd induction, CASPAR (LOC106673865) – a negative regulator of the IMD pathway – was suppressed as was PELLINO (LOC106670316), an E3 ubiquitin ligase associated with signal amplification in the pathway, hinting at their roles as essential regulatory nodes or feedback checkpoints. Furthermore, the unique expression trajectory of POSH (LOC106672599), an E3 ubiquitin ligase known to regulate both Toll and Imd pathways in the midgut/Gr– sample, implies a nuanced role in modulating the Imd response during bacterial interactions.

#### 3.3.7 Modulation of redox genes

As shown in *D. melanogaster*, exposure to bacteria prompts multi-faceted cellular responses, including the management of oxidative stress (86). A suite of redox genes appears to become finely tuned upon encounter of bacterial pathogens (87). We have mined redox-related genes in sample comparisons (**Supplementary Table S4**). Comparing midgut/Gr– to midgut/control samples revealed two dual oxidases (DUOX) genes (LOC106661409 and LOC106662103) that were up-regulated by a Log2FC factor of 1.248 and 0.451, respectively. Duox in insect guts interacts with ingested microbes by producing bactericidal reactive oxygen species (ROSs) or by creating a physical barrier. These functions in mucosal immunity are well-described (88). Moreover, upregulations of thioredoxin peroxidase TPX2 genes (LOC106662655, Log2FC 0.835; LOC106663060; Log2FC 0.508) indicate elevated hydrogen peroxide production and subsequent detoxification processes. Conversely, in midgut/Gr+ samples, despite the upregulations of DUOX (LOC106661409, Log2FC 1.321) and TPX2 (LOC106669365, Log2FC 0.701), there was significant downregulation of the HPX3 gene (LOC106661969, Log2FC -0.771) and the DBLOX-like gene (LOC106664307, Log2FC -0.967), indicating a complex balance between ROS production and management. In RoB/Gr– samples, clearly up-regulated were genes such as HPX3 (LOC106661969, Log2FC 2.524), DBLOX (LOC106661888, Log2FC 1.362), and DUOX (LOC106664686, Log2FC 0.762), suggesting a robust oxidative response to bacterial challenges. Lastly, in RoB/Gr+ samples, similar to the midgut/Gr+ samples, up-regulated genes were DUOX (LOC106665241, Log2FC 1.363) and TPX2 (LOC106674075, Log2FC 1.157), and down-regulated was the DBLOX-like gene (LOC106664307, Log2FC 0.991).

#### 3.3.8 Investigation of apoptosis, autophagy, RNA interference, serine proteinases, stress responses, and TEP processes in the midgut and RoB upon bacterial exposure

Apoptosis, autophagy, RNA interference (RNAi), serine proteinases, stress responses, and thioester-containing proteins (TEPs) all underpin insect immune defenses. They orchestrate cell fate, recycle cellular components, silence foreign nucleic acids, facilitate immune signaling, adapt to environmental challenges, and serve innate immunity roles, thereby ensuring that insects effectively manage infections and maintain resilience against pathogens. The specific genes underlying these immune responses in bed bugs remain unexplored (5, 22, 33, 35, 44, 86, 87, 89). We investigated gene regulatory patterns associated with apoptosis and the cysteinyl aspartate protease (CASP) gene families, as well as cellular processes including autophagy, RNAi pathways, serine proteinases, stress responses, and TEPs in both the midgut and RoB following ingestion of bacteria (refer to **Supplementary Table S4**). Our data show significant upregulation of specific genes in these pathways. Notably, there is compelling evidence for the activation of the RNAi pathway. This activation likely represents a defensive mechanism, wherein the midgut generates silencing RNAs (siRNAs) to combat bacterial infections. A more comprehensive exploration, such as determining the siRNAs or microRNAs (miRNAs) that are present, and their specific targets after bacterial exposure, will further elucidate the intricate context-specific role of RNAi.

### 3.4 Enriched gene ontology

**Figure 5:**
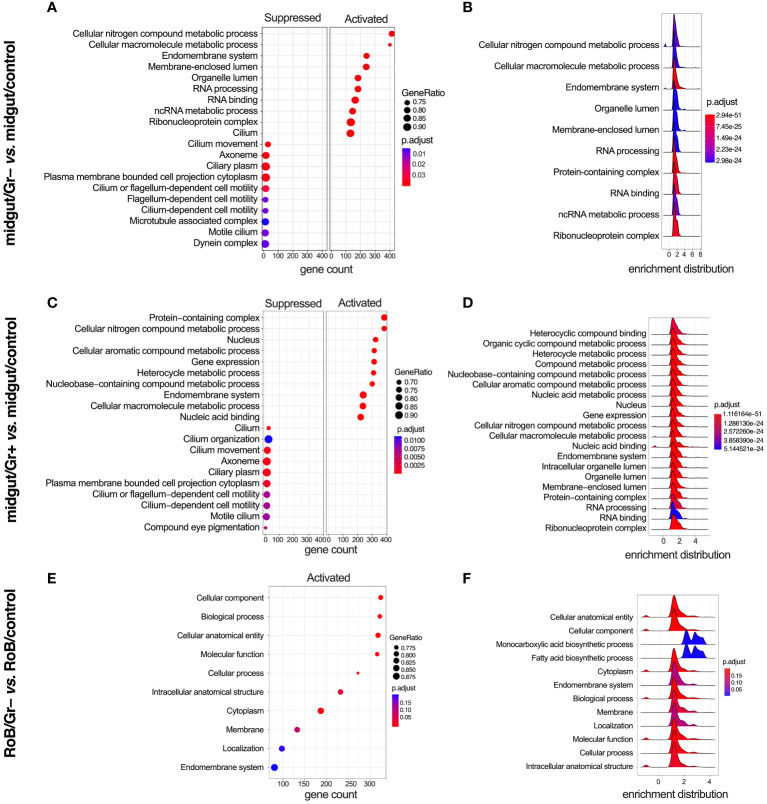
Gene ontology (GO) enriched terms in midgut and RoB (Rest of Body: body minus head and midgut tissues) samples obtained from male adult bed bugs after ingestion of sterile blood (control treatment) or blood laced with the Gram-negative (Gr–) bacterium *Escherichia coli* K12/D31 or the Gram-positive (Gr+) bacterium *Bacillus subtilis* subsp. *spizizenii* ATCC 6633. **(A, C, E)** Dot plots showing GO terms from biological processes, molecular functions and cellular components ranked according to gene count. Gene ratio (% of total DE genes in a given GO term) and adjusted p-value are also plotted. **(B, D, F)** Ridgeplots showing the density of Log_2_ Fold Changes (Log_2_FCs) for core genes in each term.

**Figure 6:**
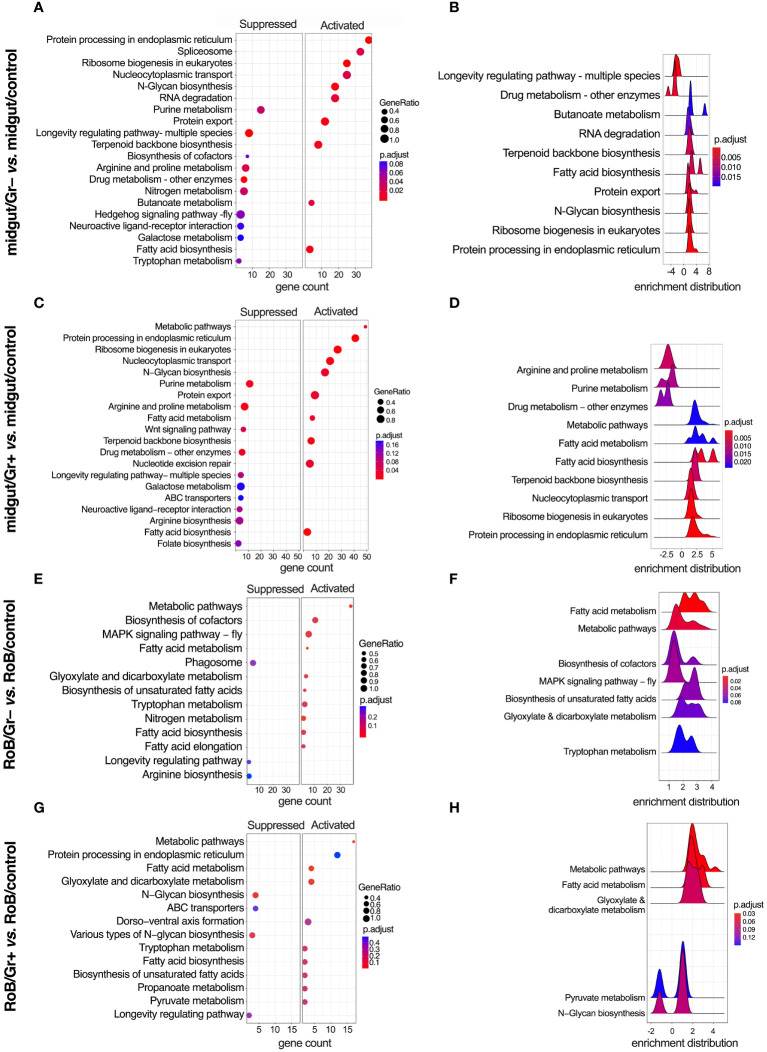
KEGG (Kyoto Encyclopedia of Genes & Genomes) enriched terms in midgut and RoB (Rest of Body: body minus head and midgut tissues) samples obtained from male adult bed bugs after ingestion of sterile blood (control treatment) or blood laced with the Gram-negative (Gr–) bacterium *Escherichia coli* K12/D31 or the Gram-positive (Gr+) bacterium *Bacillus subtilis* subsp. *spizizenii* ATCC 6633. **(A, C, E, G)** Dot plots showing KEGG terms from biological processes, molecular functions and cellular components ranked according to gene count. Gene ratio (% of total DE genes in a given KEGG term) and adjusted p-value are also plotted. **(B, D, F, H)** Ridgeplots showing the density of Log_2_ Fold Changes (Log_2_FCs) for core genes in a term.

We analyzed enriched GO and KEGG (Kyoto Encyclopedia of Genes & Genomes) terms in all pairwise comparisons (**Figures 5**, **6**). There were enriched GO terms in midgut/Gr+ and midgut/Gr– samples relative to midgut/controls (**Figures 5A–D**), and in RoB/Gr– samples relative to RoB/controls (**Figures 5E, F**) and other comparisons (**Supplementary Table S5**). No significantly suppressed GO terms were found in RoB/Gr+ samples relative to RoB/controls. In the midgut, striking parallels emerged in response to both Gr+ and Gr– bacteria (**Figures 5A–D**). Key cellular and molecular pathways displayed consistent modulation, emphasizing the possibility of conserved immune responses in these insects. Terms associated with core cellular machinery like ‘Endomembrane system’ and ‘RNA processing’, along with different metabolics, such as ‘Cellular nitrogen compound metabolic process’, were ubiquitously up-regulated regardless of the type of bacteria (**Figure 5**). Conversely, structural components of the cell, including ‘Cilium movement’ and ‘Cilium-dependent cell motility’, were consistently down-regulated (**Figures 5A, C**).

In RoB/Gr– samples, several pathways and terms predominantly associated with lipid dynamics and cellular secretions, such as ‘Cytoplasm’, ‘Endomembrane system’, and ‘Fatty acid biosynthesis’, were uniformly up-regulated in the wake of exposure to Gr– bacteria (**Figures 5E, F**). The enrichment map for the midgut shows three main GO clusters related to protein localization, protein metabolism, and various hydrolase activities (**Supplementary Figure S1**). Up-regulated and down-regulated GO terms associated with other comparisons, such as midgut/control *vs.* RoB/control, midgut/Gr– *vs.* RoB/Gr–, and midgut/Gr+ *vs.* RoB/Gr+, are represented in **Supplementary Table S5**. There were no enriched GO terms in comparisons of RoB/Gr+ *vs.* RoB/Gr– samples.

### 3.5 Conserved KEGG pathways

KEGG-enriched pathways are illustrated in **Figure 6** and in **Supplementary Figures S1**, **S2**. After bed bugs ingested Gr+ or Gr– bacteria, overlapping response patterns emerged in the midgut. Notably, several pathways related to core cellular functions, protein turnover, and metabolism, such as ‘protein processing in the endoplasmic reticulum’, ‘ribosome biogenesis in eukaryotes’, ‘N-Glycan biosynthesis’, ‘Protein export’ and fatty acid-related pathways were consistently up-regulated after exposure to both types of bacteria (**Figures 6A–D**). This overlap in response to bacteria exposure indicates heightened cellular activity, especially concerning protein synthesis and modification. Conversely, pathways associated with ‘purine metabolism’, ‘longevity-associated pathways’, and ‘arginine and proline metabolism’ were commonly down-regulated in response to ingested bacteria (**Figure 6**). Metabolic pathways centered around amino acids were either up- or down-regulated depending on the specific role of the pathway and the type of bacteria.

Ingestion of Gr+ or Gr– bacteria caused upregulation of genes in the RoB related to metabolism, protein processing, and transport, such as ‘metabolic pathways’, ‘protein processing in endoplasmic reticulum’, ‘fatty acid metabolism’, and ‘fatty acid biosynthesis’ (**Figures 6E–H**). Conversely, in the RoB, ‘longevity regulating pathway’ was down-regulated in response to both types of bacteria (**Figures 6E–H**). The enrichment map for the midgut and RoB is represented in **Supplementary Figure S2**. KEGG pathway plots were also assessed in these comparisons (**Supplementary Table S5**): midgut control *vs*. RoB control; midgut/Gr– *vs*. RoB/Gr–; midgut/Gr+ *vs*. RoB/Gr+; and midgut/Gr+ *vs*. midgut/Gr–. Notably, no enriched pathways were identified in comparisons of RoB samples (RoB/Gr+ *vs*. RoB/Gr–). These comprehensive pathway analyses unveil a multi-faceted, and possibly conserved, response strategy in the bed bugs’ midgut and RoB when challenged with our test bacteria, implying an intricate balance of metabolic and cellular adaptations to mediate immune responses.

Our study reveals a metabolic pivot in immune-challenged bed bugs, and substantiates similar phenomena reported in other insect studies (49, 51). When bed bugs ingested bacteria-laced blood, GO and KEGG terms related to metabolic pathways changed significantly. For example, longevity-associated pathways in the midgut were down-regulated irrespective of the type of bacteria used and other metabolic processes increased, including fatty acid biosynthesis and metabolism. The longevity-associated pathways that were down-regulated are often part of insulin/insulin-like growth factor signaling (IIS) and Target of Rapamycin (TOR) mechanisms, particularly in model organisms like *D. melanogaster* (90–93). These systems generally govern growth, development, and lifespan, and typically are down-regulated during infections to conserve energy for immunological defenses (90–93). Furthermore, the activation of the immune system often enhances the production of phospholipids in the endoplasmic reticulum, an organelle fundamental to the synthesis and secretion of antimicrobial peptides (51). This shift in lipid metabolism serves to support immune responses (51). Our findings support the observed metabolic responses to infections reported across insect species. Our data further highlight the critical role of the bed bugs’ midgut as an immune-related organ (69, 86, 94).

This is the first whole-transcriptome study on bed bugs in response to ingestion of sterile and bacteria-laced blood. Our data show that ingestion of blood infected with Gr+ or Gr– bacteria triggers a strong innate immune response in both the bed bugs’ midgut and RoB tissue. The midgut is the primary tissue that deals with incoming blood meals, and produces and secretes most of the digestive enzymes. It is the first site of digestion and the first site of defense against ingested pathogens. The fat body, on the other hand, is involved in nutrient storage, synthesis and transport of biomolecules, vitellogenesis, and the detoxification of dietary metabolites (49, 86, 95). The fat body is also the main immune organ in insects, producing AMPs and regulating cellular and humoral immune responses (49, 86, 95).

In our study, the immune responses in the bed bugs’ midgut and RoB differed, probably related to the mode of bacterial entry into the insects. When bed bugs ingested sterile blood, the midgut had higher transcription levels of AMP-related genes than the RoB. When bed bugs ingested bacteria-infected blood, immune-related genes were strongly up-regulated in both the midgut and RoB, but there were more DE genes in midgut than in RoB samples. Moreover, ingestion of Gr– bacteria produced more DE genes in both tissue types than ingestion of Gr+ bacteria. All data combined suggest that the midgut is more responsive to blood-fed bacterial infection than the RoB and presents a stronger basal barrier to blood feeding-related infections. In particular, intake of blood with Gr+ or Gr– bacteria triggered both Toll and Imd pathways, and prompted the production of AMPs such as defensins and prolixicins. Particularly noteworthy, the Caspar gene – which inhibits immune responses – was down-regulated in response to both kinds of bacterial infection tested. Down-regulation of Caspar affords increased resistance to bacterial infections in bed bug hemocytes (96).

Our results complement previous findings obtained with male bed bugs (94) that Gr+ bacteria trigger more sustained AMP expression than Gr– bacteria. Our results also show that Toll and Imd pathways are activated by *B. subtilis* and *E. coli*, indicating cross-talk and cross-activation of the main innate immune pathways in bed bugs. In each comparison between tissue types and treatment conditions, whether the blood ingested was sterile or bacteria-infected, a considerable portion of the most DE genes remained unidentified and uncharacterized. This lack of identification of many transcripts is common in transcriptome studies; the sialome of bed bugs features a high concentration of as-yet unidentified proteins (31). Collectively, these unidentified genes and proteins represent an intriguing area for future scientific exploration. Furthermore, whereas our study reveals the immune response 12 h post infection, further investigations at additional time points are needed to understand immune responses over time.

In conclusion, our transcriptomic analyses have shed light on the intricate interplay between midgut and RoB tissues of male bed bugs after ingestion of Gr– and Gr+ bacteria. We observed a high degree of shared gene expressions but also found distinct differences, notably in immune-related genes and metabolic pathways. These expression changes imply a multi-faceted strategy for immune defense, energy conservation, and resource reallocation, underscoring the complex physiology of bed bugs in response to pathogens. Basal expression levels of immune-related genes were higher in the midgut than in RoB tissues of male bed bugs, and ingestion of bacteria further activated their innate immune responses. This activation involves intricate cross-talk between and among various immune pathways. This study expands our current understanding of the bed bugs’ responses to pathogens and emphasizes the need to identify and characterize the numerous unidentified DE genes that are activated in response to the ingestion of bacteria. These unidentified genes and proteins present exciting new avenues for future research and may help clarify why bed bugs are the only major blood-feeding pest of humans that is not responsible for the transmission of vector borne parasites and pathogens and the diseases they cause.

## Data availability statement

The datasets presented in this study can be found in online repositories. The names of the repository/repositories and accession number(s) can be found in the article/**Supplementary Material**.

## Author contributions

SM: Conceptualization, Data curation, Formal analysis, Methodology, Investigation, Software, Validation, Visualization, Writing – original draft, Writing – review & editing. NS: Data curation, Formal analysis, Validation, Methodology, Writing – original draft, Writing – review & editing. CL: Conceptualization, Supervision, Validation, Writing – original draft, Writing – review & editing. GG: Conceptualization, Funding acquisition, Supervision, Project administration, Resources, Validation, Writing – original draft, Writing – review & editing.
