# Supplementary material for: Activation of immune pathways in common bed bugs, Cimex lectularius, in response to bacterial immune challenges - a transcriptomics analysis
Source: Front Immunol. 2024 Apr 17;15:1384193. doi: 10.3389/fimmu.2024.1384193 (PMC11061471; doi:10.3389/fimmu.2024.1384193)
Supplement: Supplementary file 1 [file DataSheet_1.docx]

Supplementary Material

# Supplementary Data

## Supplementary Figures

##
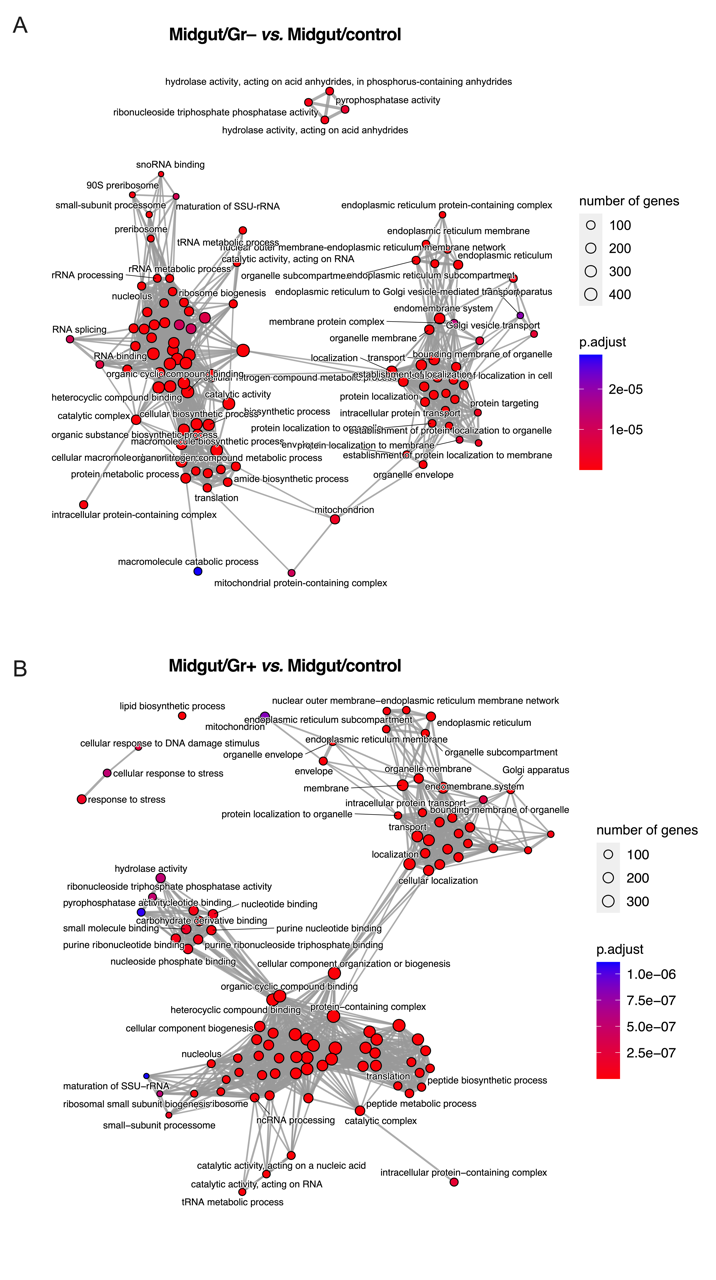


**Supplementary Figure 1**. (**A**, **B**) Enrichment Map Plots (Emplots) of Gene Ontology (GO) categories in midgut tissue samples obtained from adult male bed bugs following ingestion of blood laced with either the Gram-negative (Gr–) bacterium *Escherichia coli* K12/D3 (**A**) or the Gram-positive (Gr+) bacterium *Bacillus subtilis* ATCC 6633 (**B**). Each node corresponds to a distinct gene ontology category, with the node size indicating the number of genes in that category. Edges between nodes signify gene set overlaps. The color gradient of each node reflects the adjusted p-value, with lighter shades denoting greater statistical significance.

**
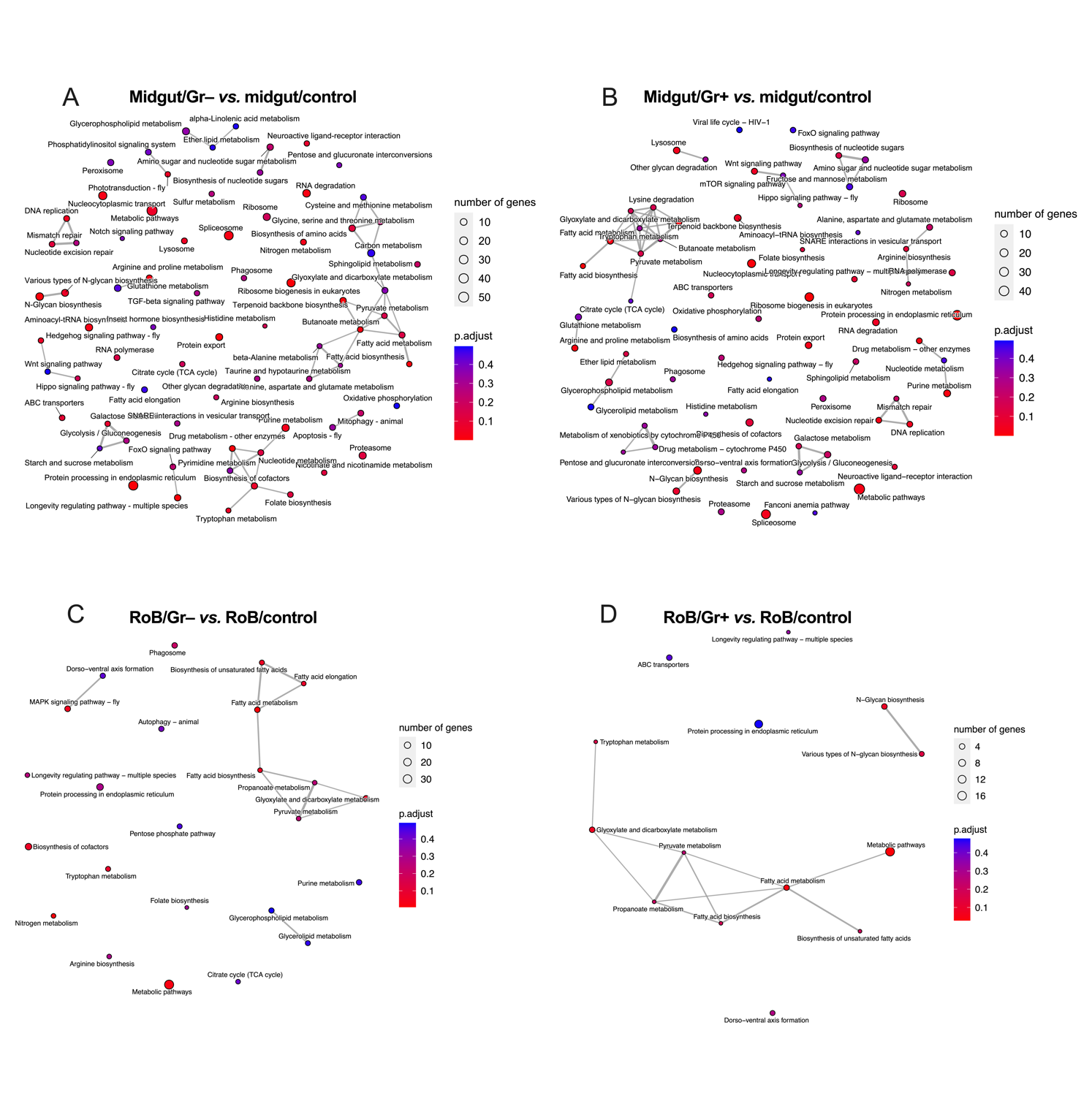
Supplementary Figure 2.** (**A**-**D**) Enrichment map Plots (Emplot) of KEGG pathways in midgut and rest of body (RoB: body minus head and midgut tissue) samples obtained from male adult bed bugs after ingestion of sterile blood (control treatment) or blood laced with the Gram-negative (Gr–) bacterium *Escherichia coli* K12/D31 or the Gram-positive (Gr+) bacterium *Bacillus subtilis* ATCC 6633. In each panel, nodes correspond to distinct KEGG pathways, and their size signifies the number of genes associated with each pathway. Edges between nodes indicate overlaps in gene sets within those pathways. The color gradient of each node reflects the adjusted p-values, with lighter shades indicating greater statistical significance.
